# Evaluation of the safety and quality of Brazil nuts (*Bertholletia excelsa*) using the tools of dna sequencing technology and aflatoxin profile

**DOI:** 10.3389/fnut.2024.1357778

**Published:** 2024-04-11

**Authors:** Victor J. R. Esperança, Paula I. O. Moreira, Davy W. H. Chávez, Otniel Freitas-Silva

**Affiliations:** ^1^Food and Nutrition Graduate Program, Federal University of State of Rio de Janeiro (PPGAN/UNIRIO), Rio de Janeiro, Brazil; ^2^Post Graduate Program in Food Science and Technology, Federal Rural University of Rio de Janeiro, Seropédica, Brazil; ^3^Embrapa Food Technology, Office of Research and Development (Sector: Operational Units – Plan V), Rio de Janeiro, Brazil

**Keywords:** microbiome, agrobiodiversity, Amazon, extractivism, rainforest

## Abstract

**Introduction:**

Brazil nuts (BNs) result from sustainable extraction and are widely exploited in the Amazon region. Due to the production characteristics in the forest and the nutritional characteristics of these nuts, the occurrence of fungal contamination and the presence of aflatoxins are extensively discussed in the literature as a great aspect of interest and concern. This study aims to evaluate the microbial profile through DNA sequencing and amplification of 16S and ITS genes for bacterial and fungal analysis, respectively, and the presence of mycotoxins using high-performance liquid chromatography with fluorescence detection (HPLC-FD) from different fractions of the nuts processed.

**Methods:**

The BN samples, harvest A (HA) and harvest B (HB), from two different harvests were collected in an extractive cooperative in the Amazon region for microbiological analysis (from DNA extraction and amplification of 16S genes, bacteria analysis, and ITS for fungi) and mycotoxins (aflatoxins AFB1, AFB2, AFG1, and AFG2) using HPLC-FD/KobraCell^®^.

**Results and discussion:**

The samples showed a very different microbiome and aflatoxin profile. Genera such as *Rothia* (HA) and *Cronobacter* (HB) were abundant during the analysis of bacteria; as for fungi, the genera *Aspergillus, Fusarium, Penicillium*, and *Alternaria* were also considered prevalent in these samples. Soil microorganisms, including those pathogenic and related to inadequate hygienic-sanitary production practices, as well as aflatoxins, were found in the samples. However, they were within the established limits permitted by Brazilian legislation. Nuts have a diverse microbiota and are not restricted to fungi of the genus *Aspergillus*. The microbiological and toxicological profile can vary significantly within the same nut in the same extraction region and can be exacerbated by global climate changes. Therefore, it is necessary to advance sanitary educational actions by applying good production practices and inspection programs to ensure the sustainability and quality of the BN production chain.

## 1 Introduction

“Brazil nut,” “Pará nut,” or “Amazon nut” is the name popularly given to *Bertholletia excelsa*; these are fruits from nut trees sourced sustainably through extraction located in the Amazon region (1). Due to its nutritional content and the characteristics of the tropical forest in which it is found, with a hot and humid climate, this fruit becomes a suitable material for the growth of microorganisms, mainly fungi of the genus *Aspergillus flavus* (2).

Among the mycotoxin-producing fungi in BN, aflatoxins (AF) are observed with greater concern, as they cause disease and can lead to death (3). AF can cause harmful effects such as immunosuppressive, carcinogenic, mutagenic, and teratogenic effects, even in small amounts in food (4). Because they are microscopic substances that do not deteriorate the appearance of the food, it is difficult to detect them just by looking at the fruit. Studies point to the existence of four different types of aflatoxins common in Brazil nuts, which are AFB1, AFB2, AFG1, and AFG2 (1, 5).

Most of the places where these fruits are extracted are precarious and have poor hygiene conditions. For this reason, a sanitary orientation program and constant training in good production and manufacturing practices are necessary to guarantee the quality of the food (6). Health education, together with good production and manufacturing practices, is essential for the efficiency and safety of food products, being an important part of the process and a mandatory item within food safety legislation (5, 7, 8).

Ensuring a safe product with AF aflatoxin contamination within the limits of legislation is essential and a significant challenge for the productive and industrial chain, especially for BNs (7). In this sense, the relevance of this study is based on the use of advanced biomolecular techniques to evaluate the microbial profile through DNA sequencing and amplification of 16S and internal transcribed spacer (ITS) genes for bacterial and fungal analysis, respectively, and the presence of mycotoxins using HPLC-FD from different fractions of the nuts processing. Since the literature is often restricted to the genus *Aspergillus*, little attention is given to the microbial profile and the variation in its population over the production seasons.

## 2 Materials and methods

### 2.1 Samples

Samples were collected from an extractive cooperative in the Amazon region from two different harvest seasons (2022 and 2023), namely, Harvest A (HA) and Harvest B (HB). For the microbiological and mycotoxin analysis, the Brazil nut samples were separated into four fractions of shell, oil, kernel, and defatted cake, generating eight samples, namely, Shell_A, Oil_A, Kernel_A, Cake_A, Shell_B, Oil_B, Kernel_B, and Cake_B.

### 2.2 Microbiological and bioinformatic analysis

The samples were manually homogenized for microbiological detection, and an aliquot of 1 ml or 250 mg was used for total DNA extraction. The 16S genes (for bacterial analysis) and internal transcribed spacer (ITS) genes (for fungal analysis) were amplified from this DNA. The obtained PCR products were quantified using the Qubit dsDNA HS kit (Invitrogen) and sequenced using the 500V2 Sequencing Kit (Illumina) and the Illumina MiSeq Sequencer^®^ (5200 Illumina Way, Inc., CA, United States). The DNA sequences were analyzed using the Quantitative Insights into Microbial Ecology^®^ (QIIME) platform, 1.8.0 version (9). These results were compared with the literature to determine the phenotypic characteristics of the microorganisms found.

The analysis involved assessing the biodiversity and abundance of microorganisms present in the sample, as well as inferring the functions of the organisms based on the genera found. DNA extraction, 16S, and ITS amplicon library sequencing were performed on the Illumina MiSeq platform (PE 250b, ~30,000 reads). The sequences before the analysis were grouped into operational taxonomic units (OTUs) and classified at five taxonomic levels, namely, phylum, class, order, family, and genus of bacteria and archaea. Reads with < 75% of the total amplicon length were discarded. Chimeras were identified and removed from the analysis using UCHIME v. 6.1 (10). Sequences were analyzed using QIIME, and low-quality regions (< Q30) were removed using Trimmomatic v. 3.2 (11). They were clustered at a 3% dissimilarity cutoff using UCLUST and compared against the database using PyNast (9, 12).

### 2.3 Aflatoxin quantification

Aflatoxin (AF) analyses were performed according to Ribeiro et al. (13), where AFB1, AFB2, AFG1, and AFG2 subtypes were determined using the HPLC-FD/KobraCell^®^. Previously, the nut fractions (shell, kernel, and cake), except for the oil fraction, were processed in the same proportion of ultrapure water (g/g) until obtaining a homogeneous texture. Approximately 100 g of the mixture was weighed and homogenized with 200 ml of methanol (HPLC grade), 5 g of anhydrous sodium chloride (NaCl), and 100 ml of hexane (HPLC grade). The mixture was stirred for 3 min at 800 rpm and filtered through fast filter paper and vacuum PTFE membrane filtration. Subsequently, the samples were purified and submitted to the HPLC-FD system.

Standard curve: For each of the seven curve points, solutions with varying concentrations of the four AFs were created, beginning with a certified standard solution containing the AFs. AFB1 and AFG1 analytical curve values ranged from 0.0004 to 0.030 μg/ml, whereas AFB2 and AFG2 concentrations ranged from 0.0002 to 0.015 μg/ml. Shimadzu's LC-20A chromatographic system was used to analyze AFs using the HPLC/Kobra-Cell^®^/DFL system (14). The Kobra-Cell^®^ post-column derivatizer from R-Biopharm was positioned between the fluorescence detector intake and the column outlet.

### 2.4 Parameters of chromatography

The chromatographic column used was the Waters Technologies' X-Bridge^®^ RP18 column, with a particle size of 5 μm (dimensions of 4.6 × 150 mm). The mobile phase is made up of acetonitrile, methanol, and ultra-pure water in a ratio of 15:20:65 v/v. It also contains 585 μl of a 4 M HNO_3_ solution and 198 g/L KBr. The flow rate is 0.8 ml/min in isocratic mode. The fluorescence detector is set to excitation at 360 nm and emission at 440 nm. The injection volume is 40 μl.

### 2.5 Statistical analysis

The microbiome results were expressed in terms of bacterial and fungus community relative abundances, represented as percentages. A heatmap was applied to reveal these abundances. A chord diagram was used to study the associations between bacterial and fungal abundances; the unidentified fragments were not included in the chord diagram. Principal coordinate analysis (PCoA) was carried out to understand sample dissimilarity and how the microbiome was affected in the four analyzed fractions of BNs and the two harvests. PCoA was performed after log conversion as log (abundance +1) and using the dissimilarity matrix obtained using the Bray-Curtis method.

Hierarchical clustering (HC) of PCoA results was applied to the group samples as a function of the coordinates resulting from PCoA. The correlation network was created from Pearson's correlation (r) matrix. The correlation network was performed considering r values between (−1 to −0.6) and (0.6 to 1) to avoid figure pollution. The magnitude of the correlations was considered following the empirical rule proposed by Teles et al. (15). All statistical calculations were performed using R version 4.1.3 (R Foundation for Statistical Computing, Vienna, Austria).

## 3 Results

### 3.1 Bacteria

**Figure 1:**
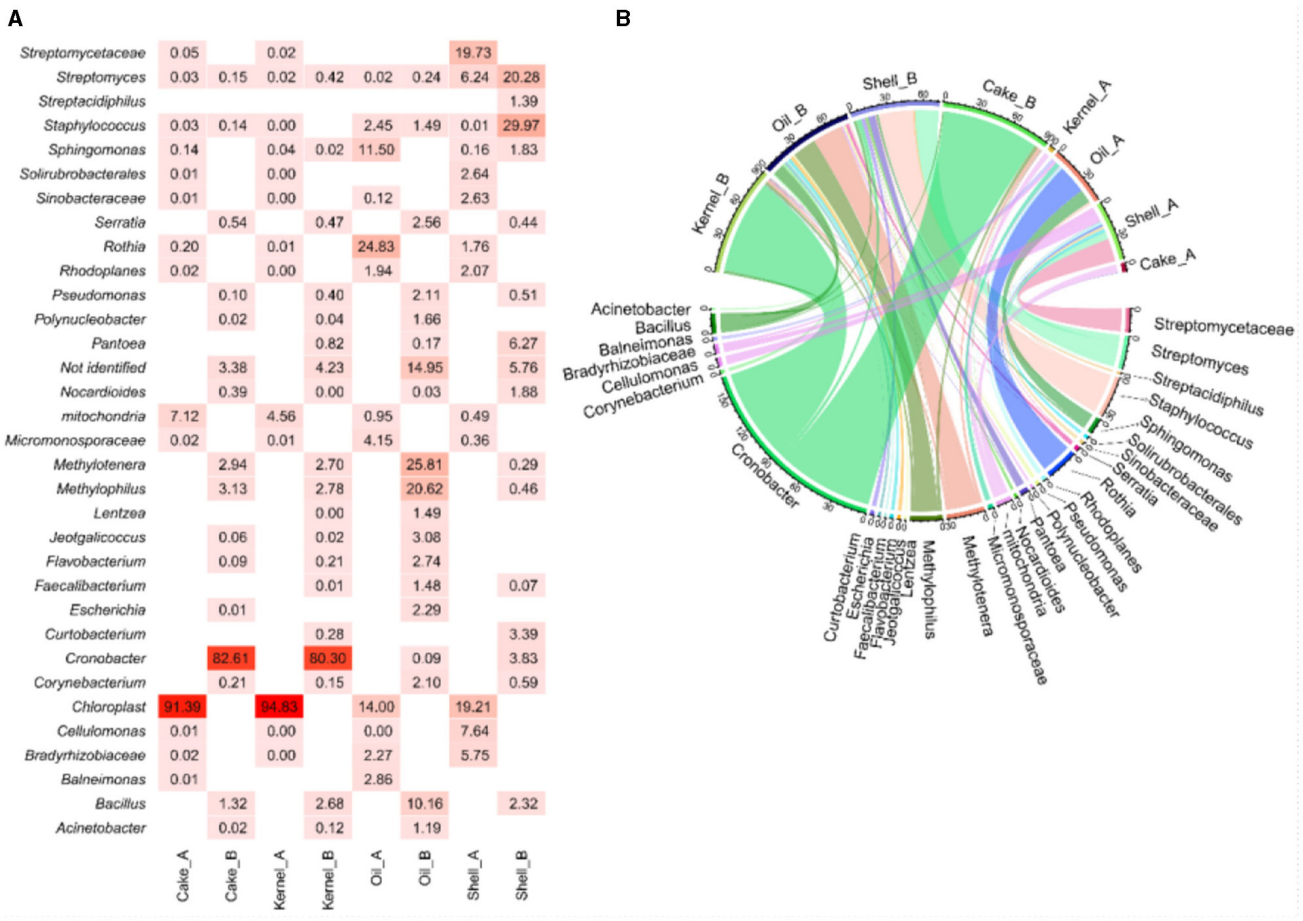
Abundance **(A, B)** chord diagram of bacteria by genus in Brazil nut fractions.

The heatmap (Figure 1A) visualized the abundance of bacterial genera, and this was complemented by the chord diagram (Figure 1B). The chord diagram is divided into two sections; the top section presents the eight samples, which are connected to the bacterial genera in the lower side. The analysis reveals that the taxa were delineated among the samples. Genera with higher abundances are represented by wider arcs, indicating a greater presence of these bacteria in the respective samples.

The Kernel_A and Cake_A samples showed chloroplast material (>90%) and mitochondrial DNA (>4%) as the most abundant material (Figure 1A). However, the same fractions from the HB harvest (Kernel_B and Cake_B) showed an intense presence of bacteria with a very similar profile between them. The most abundant genera for Kernel_B and cake_B, respectively, were *Cronobacter* (80.3% and 82.6%), which showed wide arcs (Figure 1B) and thinner arcs for bacteria of the genera *Bacillus* (2.7% and 1.3%), *Methylophilus* (2.8% and 3.13%), and *Methylotenera* (2.7% and 2.9%). For both HB fractions, a small amount of unidentified fragments were found (4.2% and 3.4%, respectively) (Figure 1A).

The Shell_A presented *Streptomycetaceae/Streptomyces* (26%), *Cellulomonas* (8%), and *Bradyrhizobiaceae* (6%) as the most abundant bacteria, followed by several genera with lesser abundance. In the HB strains, *Staphylococcus* (29%), *Streptomyces* (20%), *Pantoea* (6.3%), *Cronobacter* (3.8%), *Curtobacterium* (3.4%), *Bacillus* (2.3%), and *Sphingomonas* (2%) were identified (Figures 1A, B).

In the analysis of Oil_A, *Rothia* (24%), *Sphingomonas* (12%), *Micronosporaceae* (4%), among others with a lower percentage, *Balneimonas* (2.8%), *Staphylococcus* (2.5%), *Bradyrhizobiaceae* (2.3%), and *Chloroplasts* (14%) were identified. In this same fraction at the time, HB *Methylotenera* (25%), *Methylophilus* (20%), *Bacillus* (10%), *Jeotgalicoccus* (3.1%), *Flavobacterium* (2.8%), *Serratia* (2.6%), *Escherichia* (2.3%), *Pseudomonas* (2.1%), *Corynebacterium* (2%), and *Staphylococcus* (1.5%) were identified. The unidentified genetic material in this fraction was obtained for HB (15%), with the highest percentage from the fractions analyzed for bacteria.

**Figure 2:**
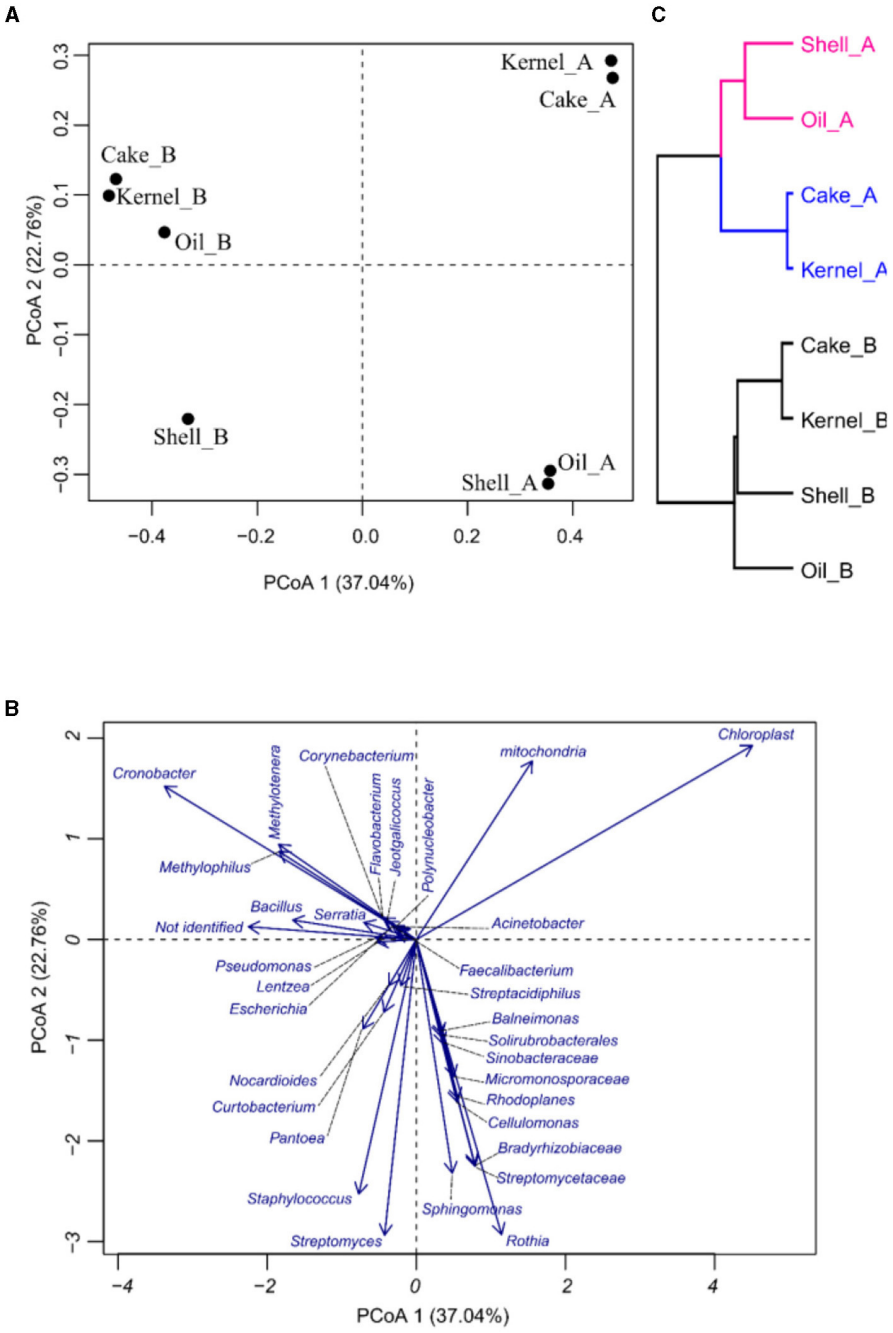
Principal coordinate analysis (PCoA) of bacteria in Brazil nut fractions, **(A)** plot for samples, **(B)** plot of bacteria, and **(C)** hierarchical clustering of principal coordinate analysis.

The PCoA was applied to see how the variability of the community was affected by the different fractions and harvests of the Brazil nut. In PCoA, the first two components explain 59.4% of the percent of variance explained (PVE), indicating a good dimensionality reduction. It is possible to observe a clear differentiation among the samples (Figure 2A). For example, Kernel_A and Cake_A were characterized by having the highest abundances of *Chloroplasts* and the lowest abundances of *Staphylococcus* (Figure 1B). On the other hand, Cake_B and Kernel_B had the most considerable abundance of *Cronobacter*, while Oil_B was represented by a greater abundance of *Methylotenera* and *Methylophilus* (Figures 2A, B). Oil_A and Shell_A were represented by *Rothia* and *Streptomyceaceae*, respectively. Finally, Shell_B had a greater abundance of *Streptomyces* and *Staphylococcus*.

The HC of the PCoA presented in Figure 2C confirmed the groups suggested by PCoA. It was divided into two harvests (HA and HB). Harvest A was divided into two groups, pointing to Kernel_A and Cake_A, containing similar communities.

**Figure 3:**
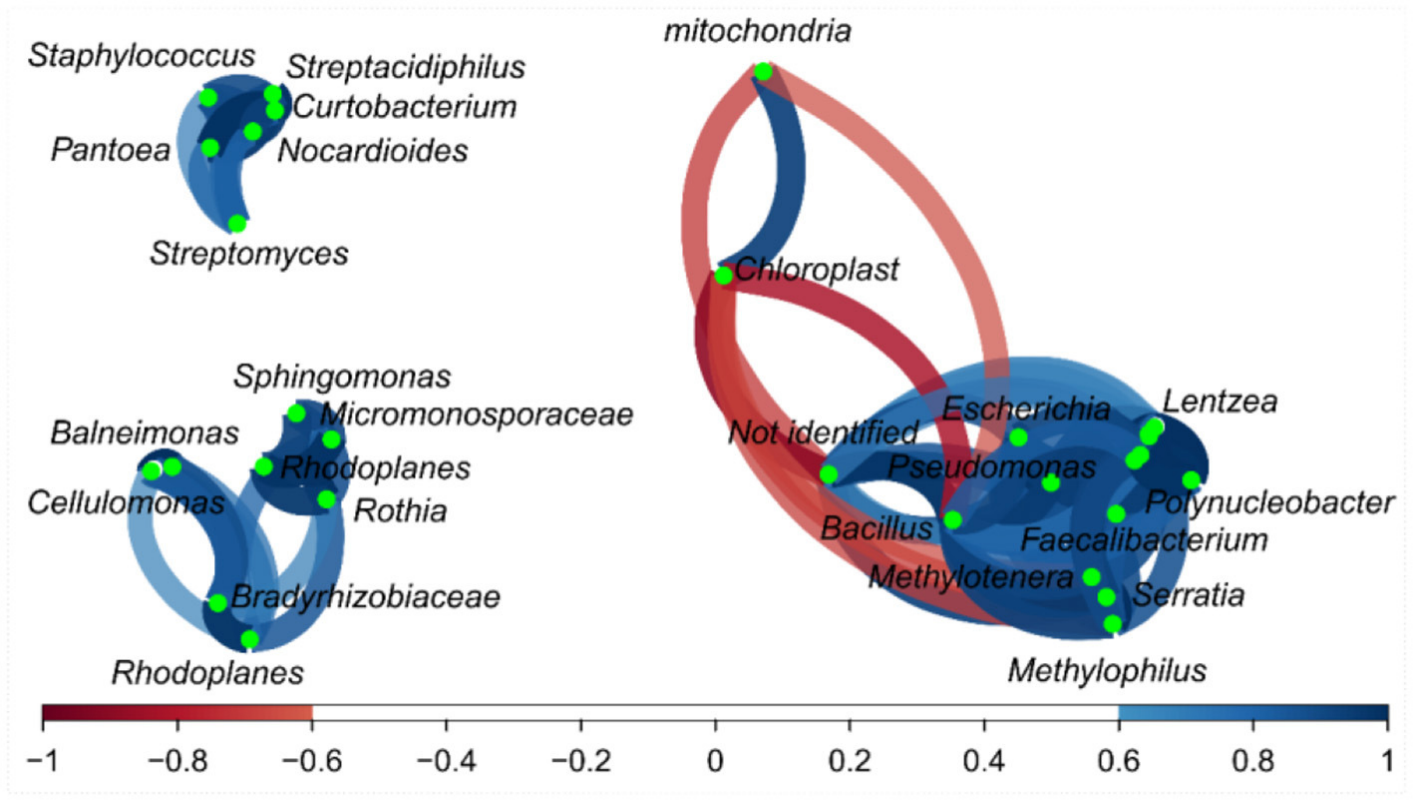
Correlation network of bacteria on the fractions from Brazil nuts. The correlation network considered r values between (−1 to −0.6) and (0.6 to 1).

Some relationships among bacterial genera in the correlation network were detected (Figure 3), and a small group (top left) was formed by bacteria such as *Staphylococcus* vs. *Streptacidiphilus* (*r* = 0.92); *Streptomyces* vs. *Streptacidiphilus* (*r* = 0.82); *Streptomyces* vs. *Solirubrobacterales* and *Sinobacteraceae* (*r* = 0.99); and *Patoea* vs. *Streptpmyces, Streptacidiphilus*, and *Staphylococus* (0.77, 0.95, and 0.86, respectively). Another group of related bacteria can be observed in the bottom left, e.g., *Sphingomonas* vs. *Micromonosporaceae* (*r* = 0.90), *Sphingomonas* vs. *Rothia* (*r* = 0.87), *Rhodoplanes* vs. *Micromonosporaceae* (*r* = 0.77), and *Cellulomonas* vs. *Bradyrhizobiaceae* (*r* = 0.83). The third group could be seen on the right side; this group has bacteria with both positive and negative correlations; *Bacillus* had a positive correlation with *Serratia, Pseudomonas, Polynucleobacter, Methylotenera, Methylophillus, Lentzea, Jeotgalicoccus, Flavobacterium, Faecalibacterium, Escherichia*, and *Corynebacterium* (r values varied from 0.77 to 0.98); and finally, the mitochondria, chloroplast, and not-identified fragments presented a negative correlation with some bacterial fragments.

### 3.2 Fungi and aflatoxins

**Figure 4:**
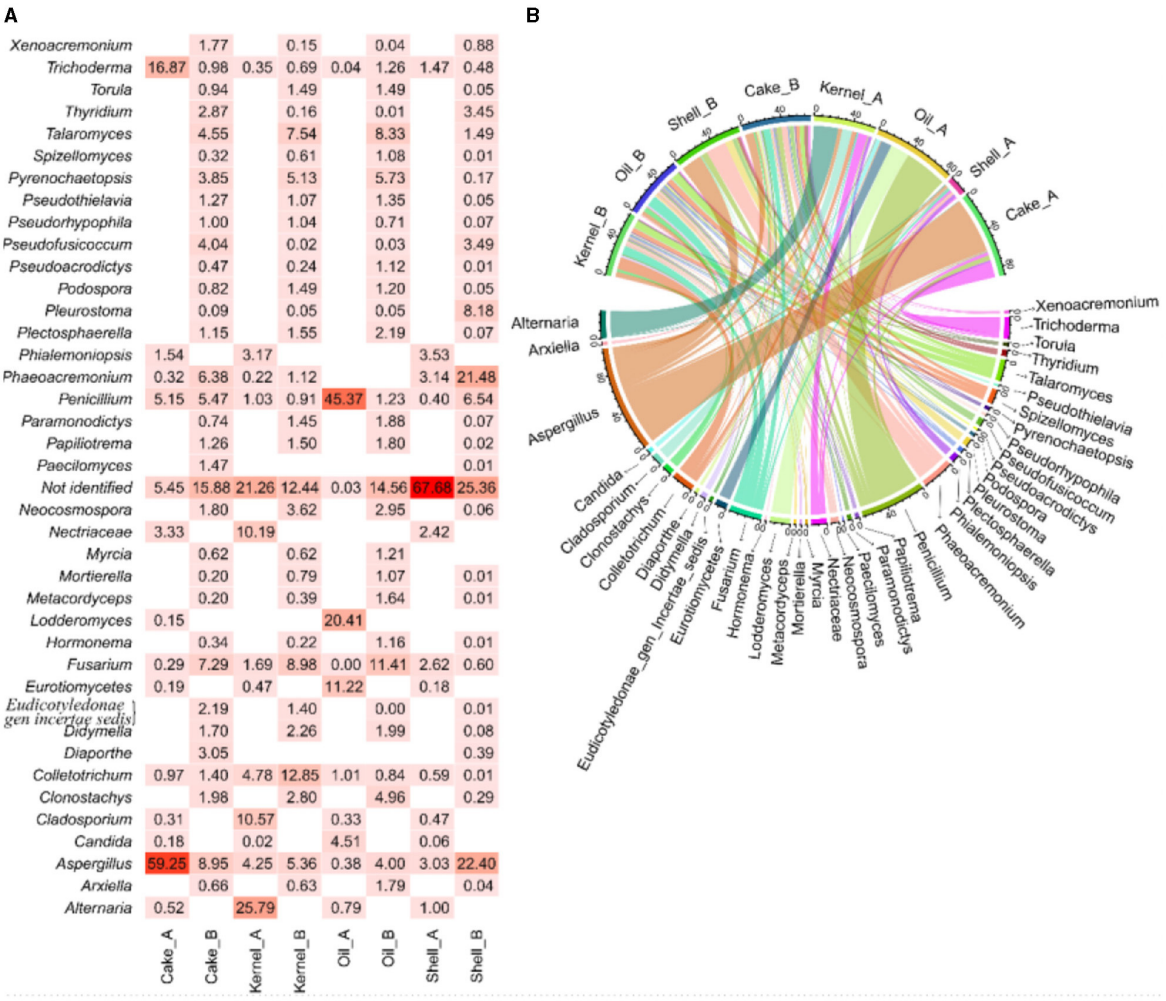
Abundance **(A, B)** chord diagram of fungi by genus in Brazil nut fractions.

The abundance of fungal genera highlights the differences between the two harvests (Figure 4A), and these differences were corroborated by the chord diagram (Figure 1B). In the chord diagram, the samples are displayed in the top part, while the fungal genera are displayed on the bottom. Taxa were delineated among the samples, with genera having wider arcs that are related to samples by the arcs.

The shell_A fraction had the highest abundance (67%) of unidentified genetic material, followed by *Aspergillus, Phaeoacremonium*, and *Phialemoniopsis* (approximately 3% each) and *Fusarium* (2.6%), and then followed by several genera with low abundance (Figure 4A). The kernel_A sample, in addition to unidentified genetic material (~22%), *Alternaria* (26%), *Cladosporium* (11%), and *Nectriaceae* (10%), presented arcs of larger bands for this sample in the chord diagram (Figure 4B). The Cake_A sample mainly presented *Aspergillus* (60%) and *Trichoderma* (10%) (Figures 4A, B). In Oil_A, the presence of *Penicillium* (24%), *Lodderomycetes* (21%), and *Eurotiomycetes* (11%) was observed, among others with lesser abundance.

Regarding harvest B, the fractions also contained genetic material that was not identified by the bank in the following proportions: Shell_B (25.4%), Cake_B (15.9%), Oil_B (14.6%), and Kernel_B (12.4%), as shown in Figure 1A. In addition, most fractions (Cake_B, Kernel_B, and Oil_B) showed a similar profile and were more balanced in their abundances (Figure 4A), resulting in more balanced arcs in width (Figure 4B), with the presence of a majority of *Fusarium filamentous fungi* (7.3%−11.4%), *Talaromyces* (4.6%−8.3%), *Aspergillus* (4.0%−9.0%), and *Pyrenochaetopsis* (3.9%−5.7%), followed by others in lesser abundances. The Kernel_B samples also included *Colletotrichum* (13%) as one of the most abundant. The Oil_B presented *Fusarium* (11.4%), followed by *Talaromyces* (8.3%) and *Pseudothielavia* (5.7%). The Cake_B sample had the highest abundance of *Aspergillus* (9%), *Fusarium* (7.3%), *Penicillium* (5.5%), *Phaeoacremonium* (6.4%), and *Pseudofusicoccum* (4.0%). The Shell_B sample differed a little, showing less *Pyrenochaetopsis* (0.2%) and *Fusarium* (0.6%) but greater abundance of *Aspergillus* (22%) and *Phaeoacremonium* (21.5%) and the presence of *Pleurostoma* (8.1%) and *Penicillium* (6.5%).

**Figure 5:**
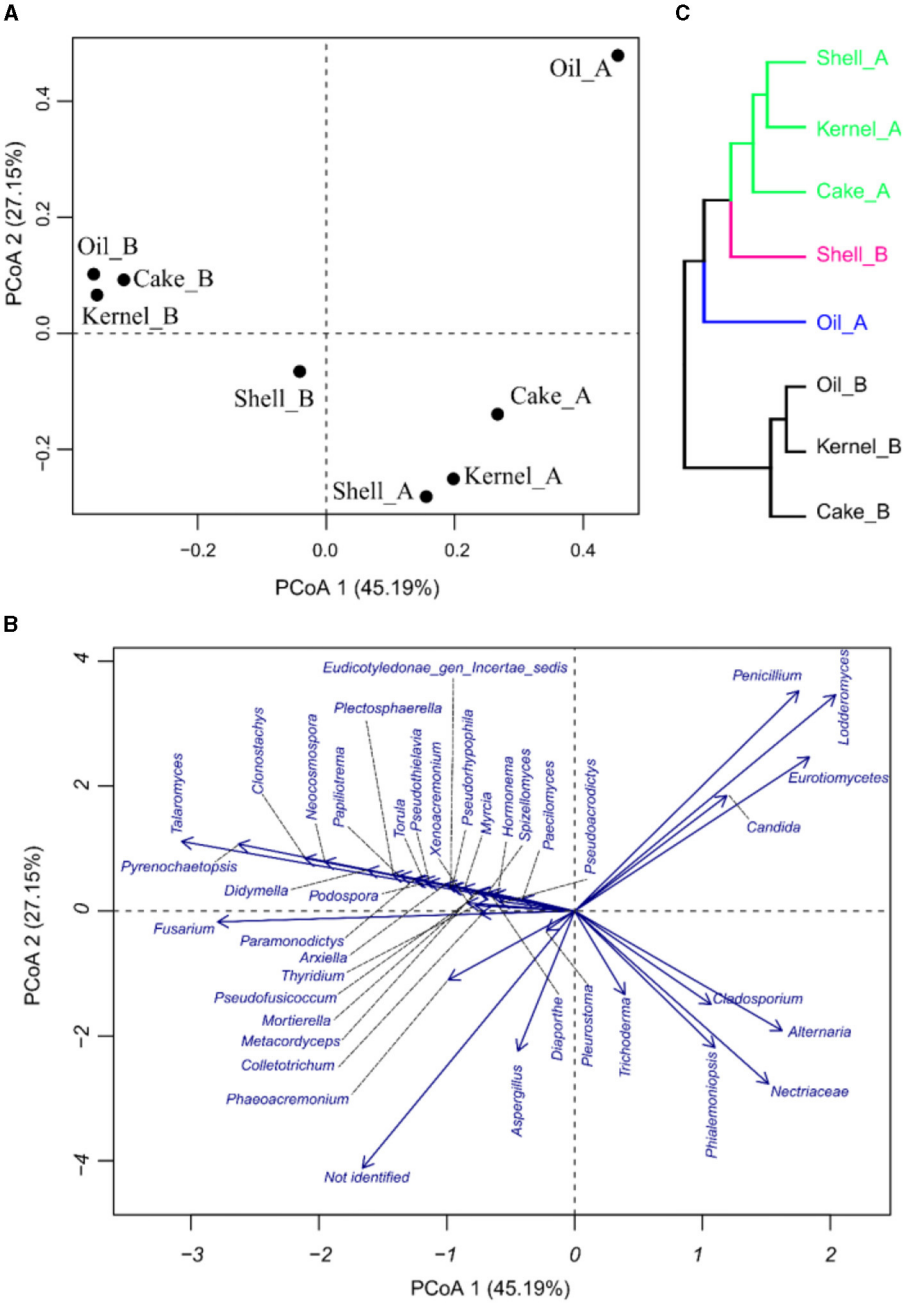
Principal coordinate analysis of fungi in Brazil nut fractions, **(A)** plot for samples, **(B)** plot of fungi, and **(C)** hierarchical clustering of principal coordinate analysis.

The PCoA results for fungal abundances allow us to observe the variability of the taxa fungus community through the fractions and harvest of Brazil nuts. The components in PCoA contain 72.34% of the PVE (Figures 5A, B), indicating a good dimensionality reduction. The samples for the two harvests were well discriminated (Figure 5A). The fractions of harvest B were close to one another, especially for Kernel_B, Oil_B, and Cake_B; these samples were characterized by higher abundances of *Talaromyces*, followed by many others with lower fungal abundances, as observed in Figure 5B. Oil_A had the highest abundance of *Penicillium, Lodderomuces, Eurotiomyces*, and *Candida*. On the other hand, the samples with higher abundances of *Aspergillus* were Cake_A, followed by Shell_B; the same samples also presented the biggest values of not-identified materials, followed by Kernel_A. Finally, the HC of the PCoA (Figure 5C) showed four sample groups, two of which contained three samples each. The green group consisted of samples Shell_A, Kernel_A, and Cake_A; the black group consisted of Oil_B, Kernel_B, and Cake_B formed. The pink and blue groups were formed by the individual samples Shell_B and Oil_A, respectively.

**Figure 6:**
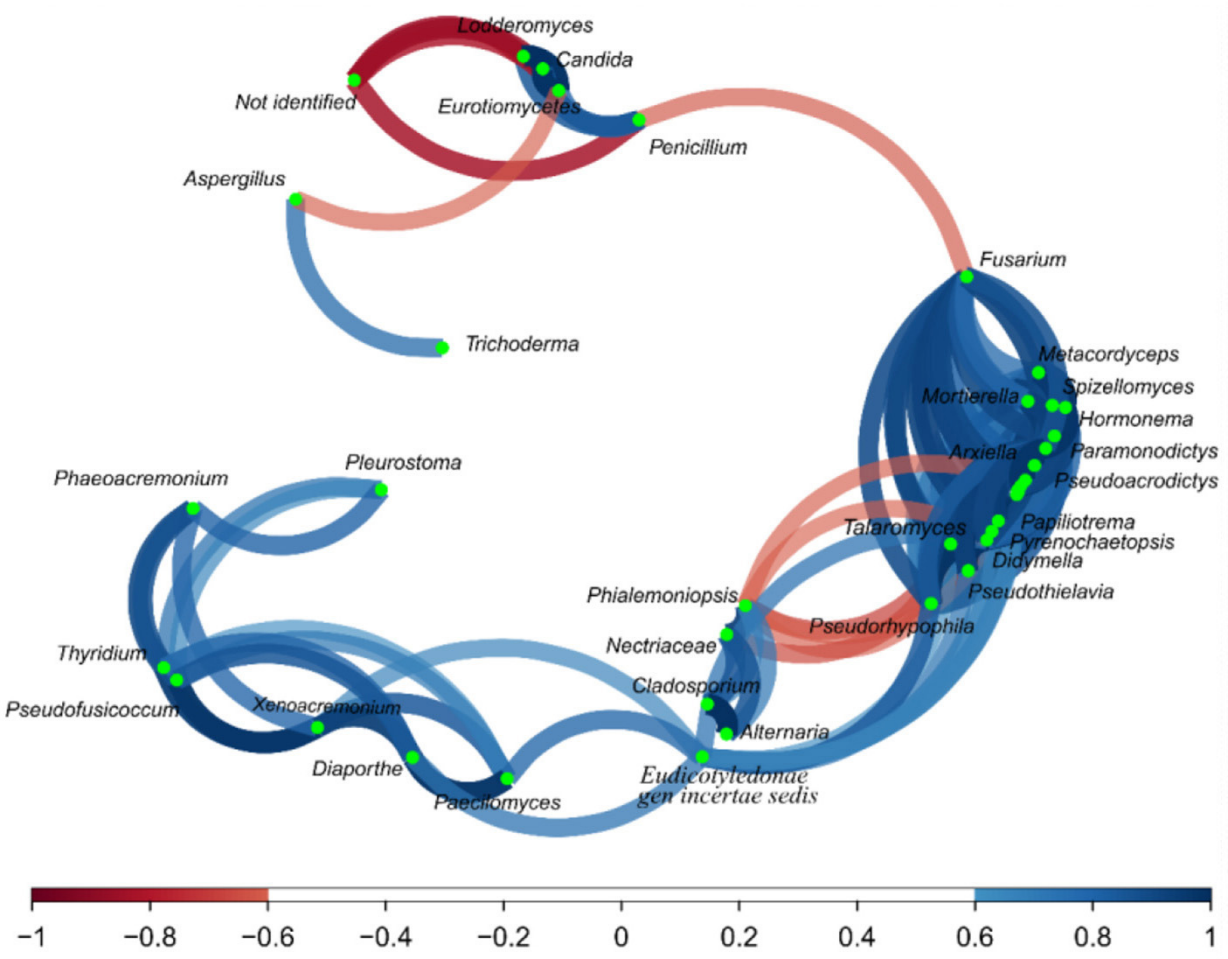
Correlation network of fungus on the fractions from the Brazil nuts. The correlation network considered r values between (−1 to −0.6) and (0.6 to 1).

Regarding the correlation network (Figure 6), *Aspergillus* and *Trichoderma* presented a strong correlation (*r* = 0.74), as did *Penicillium* vs. *Lodderomuces, Eurotiomycetes*, and *Candida*, with r values of 0.83, 0.78, and 0.83, respectively.

**Figure 7:**
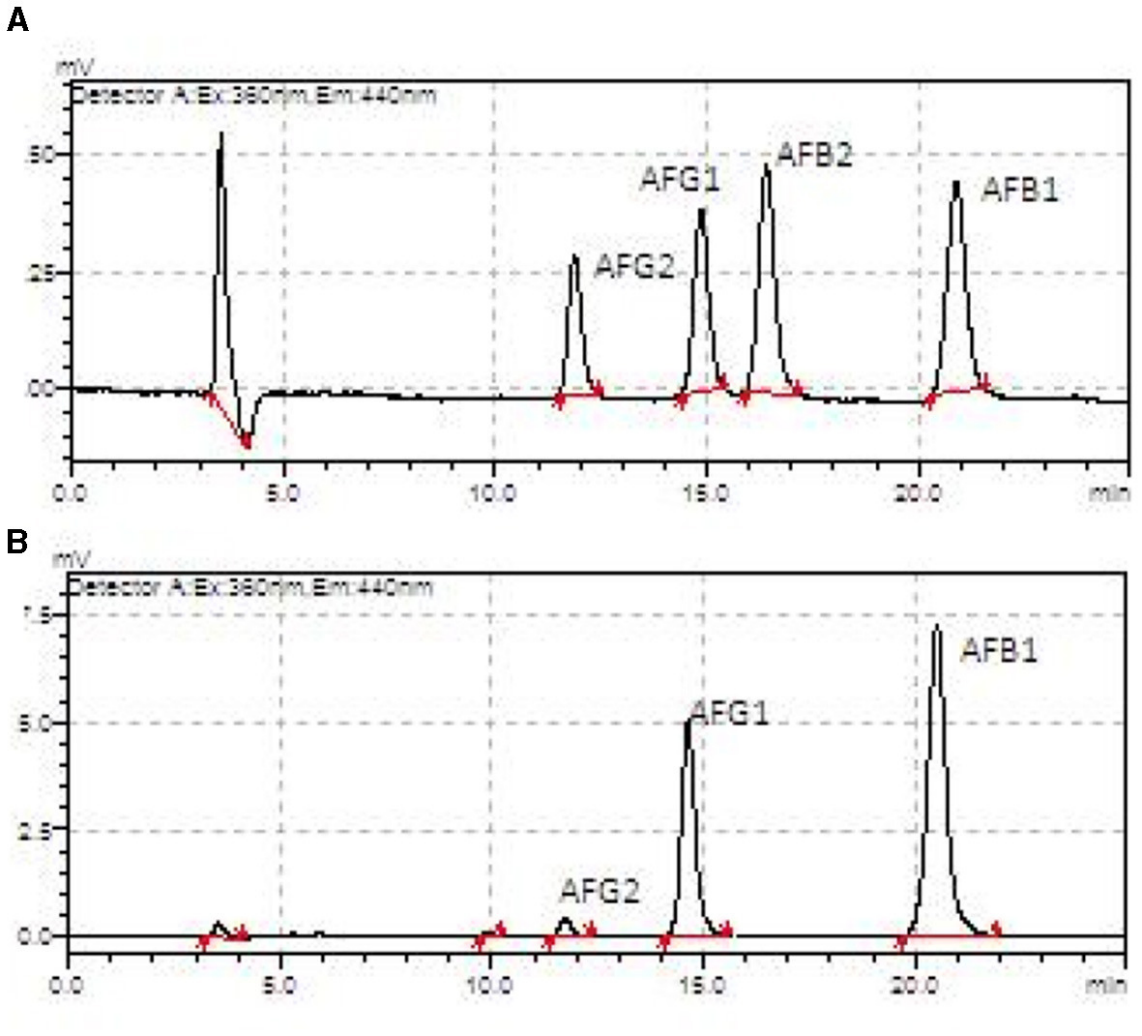
Aflatoxin chromatograms from an HPLC-fluorescence detector showing AFB_1_, AFB_2_, AFG_1_, and AFG_2_ in the Brazil nuts samples. **(A)** Oil sample from harvest A. **(B)** Oil sample from harvest B.

The aflatoxin profile was determined for the AFB_1_, AFB_2_, AFG_1_, and AFG_2_ subtypes and expressed in Table 1. The HB harvest samples, in general, presented higher amounts of most subtypes compared to the HA harvest samples, except for the kernel fraction (all subtypes), shell (AFG_1_), cake (AFB_2_), and oil (AFB_2_) (Figure 7).

**Table 1:** Results of aflatoxin subtypes identified in the HA and HB fractions.

**Harvest**	**Fraction**	**AFB1**	**AFB2**	**AFG1**	**AFG2**
A	Shell	0.43	ND	2.56	ND
B		0.80	ND	0.40	ND
A	Cake	13.46	0.41	36.42	0.50
B		47.91	ND	41.35	3.80
A	Kernel	0.71	ND	0.51	ND
B		ND	ND	ND	ND
A	Oil	5.91	0.21	9.34	0.22
B		16.66	ND	11.45	1.28

The results of the sample means are expressed in μg/kg. ND, not detected by the method.

The samples at the two collection times showed different microbiomes and aflatoxin profiles. It was observed that in HB, there was greater microbial variability. Oil and shell have the most diverse profiles among the other fractions. As for the fungus profile, HB also exhibited greater variability. However, kernel, cake, and oil were similar in the abundance of genera diversity results. The results of the aflatoxin analyses showed that the most contaminated fraction was the cake for both HA and HB.

## 4 Discussion

In the HA, the bacteria identified in Shell_A are found in different environments, mainly in soil and decomposing material, as they have saprophytic characteristics. This result was expected since this fraction is in direct contact with the external environment and consequently more contaminated (1, 6, 16). On the other hand, Kernel_A and Cake_A showed a significant reduction in these bacteria, with only plastid DNA being detected in the majority, indicating low contamination of these fractions and adequacy in the good practices of processing these fractions.

When observing the Oil_A fraction, in addition to the expected plastid DNA, the presence of a greater proportion of different bacteria was observed, such as the genus *Rothia*, which are bacteria found in human skin and saliva but which could be emerging as opportunistic pathogens associated with various infections in immunocompromised individuals, drawing attention to the study of their diagnosis and symptoms presented by patients (17). In addition, the presence of the genus *Staphylococcus* in the oil, which is usually present in the respiratory cavity in humans and can act as pathogenic bacteria (18), may indicate contamination due to improper handling of these fractions during processing (19). *Sphingomonas* are bacteria found in different environments; however, their presence in Oil_A (HA) can be problematic since these bacteria are used precisely in bioremediation because of their ability to degrade oils. Associated with this, the presence of this bacteria, as well as the presence of fungi (*Aspergillus, Fusarium*, and *Penicillium*) in the samples, can greatly contribute to the degradation of this oily matrix, generating a product with unpleasant sensory and physical-chemical qualities (20–22). In addition, some species of this genus, such as *Sphingomonas paucimobilis*, an emerging opportunistic bacterium with a particular tropism for bone and soft tissue (23); nosocomial diseases (24, 25); meningitis (26); endocarditis (27); and some cases of pneumonitis caused by *Sphingomonas ginsenosidimutans* (28). On the other hand, in harvest B, in addition to bacteria, naturally occurring in different environments, such as soil and decomposing material, others from the genus *Cronobacter* were also identified. These bacteria are Gram-negative, facultatively anaerobic, opportunistic pathogens. Several species are resistant to desiccation and persistent in dry food products. They may even contribute to the deterioration of nuts, depending on the storage conditions. This is particularly relevant since nuts are predominantly consumed in this manner (29–31). Several bacteria identified in HB also have species described as human opportunistic pathogens which, as in the case of *Pseudomonas*, cause recurrent nosocomial infections and are also an important indicator of food deterioration, which is a danger because under conditions of low immunity or favorable concentration, they can trigger serious diseases, with many species described as highly resistant to antimicrobials in traditional use in medical protocols (32–34).

This information on bacteria is relevant, as most of the literature on nuts focuses only on bacteria from the microbiological standards of food in current Brazilian legislation (35) or on the classic fungal microbiota (5, 36–39). Regarding these, pathogenic microorganisms, such as *Salmonella* spp., were not detected in both harvests. Some samples presented *Escherichia* sp. in a very low proportion, except for the Oil_B sample, which indicated an abundance of 2.29% of this genus. One hypothesis raised is that this presence may indicate contamination of this fraction due to inadequate hygienic conditions at the site, corroborating the study by Arrus et al. (36). This genus of bacteria has different strains with different pathogens, for example *E. coli*, which can cause a variety of diseases (diarrhea, diseases associated with the gastrointestinal tract, and also co-infections such as meningitis and respiratory tract), and most of them are difficult to treat (due to observations of multiresistant to antibiotics), representing a greater health risk, and are often associated with diseases transmitted by food or water (40, 41).

Although the initial expectation regarding the oil fraction would be sterility or low contamination due to the low water activity of this food matrix, the exact opposite was observed. This is due to the richness and diversity of nutrients that this fraction has, thus creating a very favorable environment for microbial proliferation (42). de Oliveira et al. (43) found a similar situation and even proposed sterilization of nut oil through ozonation without impairing its physicochemical characteristics. The oil fraction was considered critical for both HA and HB, requiring greater intervention in good practices by handlers and the equipment used for extraction, transport, and storage.

The fungi observed in the samples are common in soils, with several considered phytopathogens, such as *Alternaria* and *Cladosporium*, while the genera *Aspergillus* and *Penicillium* are considered food degraders (44). These last two are the main fungal microflora and major producers of toxins that are harmful to plants, animals, and humans, with *A. nomiae* of great interest in Brazil nuts (16, 45). Fungi of the genus *Alternaria*, despite receiving little attention in the literature on Brazil nuts, are capable of producing toxins such as alternariol (AOH), alternariol methyl ether (AME), tenuazonic acid (TeA), tentoxin (TEN), and altenuene (ALT), leading to food spoilage and posing a health risk to humans. This is a major concern for several countries in Asia and Europe (46–48). Freitas-Silva et al. (49) identified AME for the first time in Brazil nuts samples using liquid chromatography with tandem mass spectrometry (LC-MS/MS) without identifying *Alternaria* spp. in the analyzed samples. Our study showed that the DNA of *Alternaria* spp. in the two Brazil nut seasons showed that, depending on the climatic conditions, this phytopathogen/mycotoxin can constitute a potential danger for the Brazil nut.

The cake and oil fractions showed the highest amounts of aflatoxins. In the case of the cake fraction, this may be related to the higher prevalence of *Aspergillus* in this fraction; however, the same cannot be said for the oil fraction, as this had the lowest prevalence of *Aspergillus* among the fractions. This same inversion of prevalence in the proportions “fungi vs. toxins” was found and discussed by Álvares et al. (50) in nut samples from the region of the state of Acre (BR), where even without the significant presence of fungi of the *Aspergillus* type, the samples contained aflatoxins produced by them. A hypothesis that could be considered is the possible cross-contamination between the fractions at the time of extraction or preparation of the product, especially in the case of oil (Figure 2 and Table 1). Another possibility is contamination from the soil, as the presence of the fungus in the fruit is not a requirement for the production of mycotoxins (51, 52).

The amounts of aflatoxins found in this study's samples followed Brazilian legislation (35) and corroborated the results found by Álvares et al. (50). However, it is important to point out that even low, small amounts of aflatoxins can, in the long term, determine the triggering of serious diseases, especially those of the AFB_1_ type, which is considered one of the most dangerous and may result in significant alterations in DNA methylation and hormones that can increase the susceptibility to various diseases, such as cancer, in addition to its hepatotoxicity (53–55). A study in mice showed that AFB_1_ doses of 6 μg/g body weight could develop hepatocellular cancer in 90% of guinea pigs after 52 weeks (56). In this sense, it is important to point out that even with a small amount of toxin, an orientation for the nut portion that should be consumed safely is also necessary (3).

Although the Brazilian legislation (35) has exclusive indications of the content of aflatoxins for Brazil nuts and also for how this nut is presented (“with shell for direct consumption,” “shell for direct consumption,” and “without shell for further processing”), these fruits are usually sold in bulk, without industrialized packaging, and without refrigeration, which means they are outside the sanitary rigor. An alternative to this would be the need to place sanitary quality inspection and certification seals, as well as the dissemination of guidance material for cleaning these foods before consumption, since they are products that, despite being dry or toasted, can also be consumed *in natura* (6, 13, 57).

For many years, it was thought that the appearance of mycotoxins was a peculiarity of the storage and storage stages of fruits; however, studies show that their production can also occur during planting and plant growth (8, 16); therefore, hygienic-sanitary attention and care both in the stages that precede and those that proceed with extractivism must be reinforced and guaranteed (58). Another strategy that can be suggested for the control of aflatoxins, due to their low price and viability, is the early harvest of these fruits, as carried out by the study by Martins et al. (52) with specimens of peanuts where they were successful in this type of intervention or even the application of temperature-controlled packaging technologies for storage and distribution (59). The early detection of gene expression in fractions of *Aspergillus* species can be a valuable tool to predict the production of aflatoxin before its detection in Brazil nuts; however, these tools are not very accessible from a financial point of view (45).

## 5 Conclusion

Brazil nuts have a diverse microbiota, which is not restricted to fungi of the *Aspergillus* genus, as is most often the focus of most literature on nuts. Saprophytic, pathogenic, and enteric bacteria were also found in the samples in different proportions, showing a non-uniformity of the samples. Fungi of the genus *Alternaria* were identified in the analyzed samples, different from previous studies. The oil fraction presented microorganisms contrary to expectations regarding water activity and microbial proliferation. The microbiological and toxicological profile can be variable according to seasonality, extraction period, collection site, fruit handling, and processing. In addition, we must consider that health education is a valuable and necessary tool for mitigating microbial contamination. This needs to be implemented efficiently and supervised within the nut production chain. Guidance on consumption, previous care, the dangers of non-hygiene, and the correct preparation of these nuts are also necessary. Therefore, there is a need for the development of more laws, educational actions, and inspection programs, as well as the monitoring of continued studies on the fluctuation of microorganisms over the years and their correlation with climate studies. This is necessary to understand whether global climate changes are affecting this valuable nut of the Amazon rainforest.

## Data availability statement

The raw data supporting the conclusions of this article will be made available by the authors, without undue reservation.

## Ethics statement

The studies involving humans were approved by the Universidade Federal do Estado do Rio de Janeiro. The studies were conducted in accordance with the local legislation and institutional requirements. The participants provided their written informed consent to participate in this study. Written informed consent was obtained from the individual(s) for the publication of any potentially identifiable images or data included in this article.

## Author contributions

VE: Writing – original draft, Writing – review & editing, Conceptualization, Data curation, Formal analysis, Funding acquisition, Investigation, Methodology, Project administration, Resources, Software, Supervision, Validation, Visualization. PM: Conceptualization, Data curation, Formal analysis, Funding acquisition, Investigation, Methodology, Project administration, Resources, Software, Supervision, Validation, Visualization, Writing – review & editing. DC: Conceptualization, Data curation, Formal analysis, Funding acquisition, Investigation, Methodology, Project administration, Resources, Software, Supervision, Validation, Visualization, Writing – review & editing. OF-S: Writing – original draft, Writing – review & editing.
